# Effects of Two Environmental Enrichment Methods on Cognitive Ability and Growth Performance of Juvenile Black Rockfish *Sebastes schlegelii*

**DOI:** 10.3390/ani13132131

**Published:** 2023-06-27

**Authors:** Fengyuan Shen, Zonghang Zhang, Haoyu Guo, Yiqiu Fu, Dong Zhang, Xiumei Zhang

**Affiliations:** 1 East China Sea Fisheries Research Institute, Chinese Academy of Fisheries Sciences, 300 Jungong Road, Shanghai 200090, China; fyshen0817@126.com (F.S.); zdfit63@163.com (D.Z.); 2 Guangdong Provincial Key Laboratory of Marine Biotechnology, Shantou University, Shantou 515063, China; zhangzh@stu.edu.cn; 3 Fisheries College, Zhejiang Ocean University, Zhoushan 316022, China; haoyuguo@163.com; 4 The Key Laboratory of Mariculture, Ministry of Education, Ocean University of China, Qingdao 266003, China; 17860826125@163.com

**Keywords:** environmental enrichment, T-maze, cognitive ability, growth performance, *Sebastes schlegelii*

## Abstract

**Simple Summary:**

The purpose of this study was to investigate the effects of two environmental enrichment methods on the growth and cognitive ability of *Sebastes schlegelii*. The main results showed that seven weeks of environmental enrichment affected their growth performance negatively, but the growth-related indicators were affected positively. Additionally, the cognitive abilities of individuals in the enrichment groups were significantly enhanced compared with those of individuals in the control group. These phenomena revealed that the *S. schlegelii* reared in two types of enrichment environments showed stronger learning and cognitive ability. The results can provide a reference for improving the efficiency of stock enhancement projects for this species.

**Abstract:**

A widely used approach to restoring marine fishery resources is stock enhancement using hatchery-reared fish. However, artificial rearing environments, which are often lacking in enrichment, may negatively affect the cognition, welfare, and adaptive capacity to new environments of juvenile fish, thereby leading to low post-release survival rates. This study examined the effects of habitat and social enrichment on the growth performance and cognitive ability of *Sebastes schlegelii*. Following seven weeks of environmental enrichment, a T-maze experiment was conducted, and the telencephalon and visceral mass of the fish were sampled to measure the growth (growth hormone: GH; insulin-like growth factor-1: IGF-1; and somatostatin: SS) and cognitive abilities (brain-derived neurotrophic factor: BDNF; and nerve growth factor: NGF)-related indicator levels. The results indicated that, although the final body length, final body weight, and specific growth rate of both enrichment groups were lower than those of the control group, both methods of enrichment had a positive impact on growth-related factors (increased GH, increased IGF-1, and decreased SS). The enrichment groups demonstrated a stronger learning ability in the T-maze test, and the levels of BDNF and NGF in the telencephalon were significantly higher in the enrichment groups than those in the control group. Additionally, there was a significant interaction between the two enrichment methods on the NGF level. This study confirms that a more complex and enriching environment is beneficial for cultivating the cognitive abilities of cultured juvenile *S. schlegelii*, and the result can provide a reference for the improvement of the stock enhancement of this species.

## 1. Introduction

The release of hatchery-reared aquatic animals into natural waters has become a crucial measure for restoring fishery resources worldwide [1]. However, stock enhancement through releasing cultured fish has been greatly affected by high mortality rates in released fish. This has undermined the actual outcome of this practice and raised significant concerns around the world, especially for marine fish enhancement [2,3,4]. For example, billions of juvenile salmon are released into the wild each year, but less than 5% of them survive to adulthood [5]. Moreover, maladaptation, which mainly includes weak predation and anti-predation capabilities, is considered one of the main reasons causing the death of juveniles released into the wild [1]. During the juvenile stage, fish are liable to starvation and predation, which accordingly aggravates their mortality [6,7]. Many researchers ascribe the high mortality rates observed in released fish to the fact that juveniles reared in artificial environments may not be adequately equipped to survive in the wild [4]. For example, a high population density is often maintained in modern industrial aquaculture systems used for commercial rearing, and the living environment is not as intricate and uncertain as the natural environment. All the factors mentioned above may result in diminished cognitive abilities and abnormal behavioral traits in fish, ultimately resulting in low survival rates after release [8,9,10]. An important means to improve this situation is environmental enrichment. Studies have shown that a complex environment can improve cognitive ability and play an important role in shaping the nervous system of fish [3,11]. Two of the most significant factors are the heterogeneity of the physical environment and social complexity, in which social interaction can provide neural stimulation and increases the number of synaptic connections and neurogenesis, thus promoting learning [12]. The complexity of the physical environment provides a similar effect to social enrichment [1,3,8].

Spatial cognition, which is particularly important for fish, involves perceiving and storing spatial cues, and being able to retrieve this information and use learning and memory to help them modify their behavior to find a location or follow a specific route [3]. Thus, cognitive abilities are particularly important for the survival of fish inhabiting complex and changeable environments [13]. Generally, cognition can be defined as changes in animal behavior and physiology with experience [14]. Many studies have confirmed that a barren environment weakens the cognitive abilities of fish [3,15,16]. However, the influence of environmental complexity on fish cognition, both in terms of behavioral and molecular mechanisms, remains largely unknown. To better understand this relationship, it is crucial to investigate how the living environment of fish impacts their cognitive abilities through cognition assessment devices and the detection of related indicators.

In a variety of behavioral assessment methods, the maze test is widely used for evaluating the cognitive abilities of animals. Among the various types of maze tests, the T-maze is an effective method and widely used for assessing the cognitive ability of rodents [17,18], and it has also been applied to evaluate the cognitive abilities of fish in recent years [19]. In addition, neuroscientists have given special attention to the molecular mechanisms of neuroplasticity, with a particular focus on the neurotrophic factor family. Nerve growth factor (NGF) and brain-derived neurotrophic factor (BDNF), as members of the neurotrophic factor family, play significant roles in neuroplasticity. NGF, initially described as neuronal survival and growth factor, plays roles in the density of innervation, synthesis of neurotransmitters and neuropeptides, axonal sprouting, and dendritic arborization [20]. Additionally, BDNF has an important role in neural plasticity through the sculpting and refinement of synapses and through promoting neurogenesis and cell survival [21]. Both NGF and BDNF play a key role in the process of neuron proliferation, differentiation, and synaptic formation. It has been found that BDNF mRNA is widely expressed in the brain of 7-day-old zebrafish larvae, which indicates that neurotrophic factors begin to affect juveniles’ cognitive function shortly after birth [22]. As a result, they are closely related to an individual’s learning and cognitive abilities [23]. 

In addition to cognitive ability, individual size also tends to affect the predatory ability of juvenile fish and the number of parasites on the body surface, and thus its survival [24,25]. Similar to other vertebrates, the growth of fish is also endocrine-regulated, particularly through the growth hormone (GH)–insulin-like growth factor-1 (IGF-1) axis. GH is mainly secreted by somatotrophs (GH-producing cells) in the pituitary gland, and the release of somatostatin (SS) in the hypothalamus also plays a role in regulating the release of GH. At the same time, the growth hormone released from the pituitary enters the blood circulation and binds to hepatic GH receptors to stimulate the synthesis and release of IGF-1 [26]. IGF-1 is a 7.5 kDa single-chain polypeptide that is responsible for cell differentiation and proliferation, and promotes an individual’s growth by stimulating the related processes of bone growth [27].

*Sebastes schlegelii* is widely distributed in the coastal areas of Northern China, Japan, and South Korea [8]. In addition, it is an important species for stock enhancement due to its fishery value. However, its resources are declining severely due to overfishing [3,28]. *S. schlegelii* typically inhabits areas with rocks and seagrass, where it needs strong adaptability to cope with complex environmental changes and competition from other species with similar habits. In addition, *S. schlegelii* exhibits strong plasticity and adaptability, enabling it to adapt to various natural habitats [8,29]. Therefore, this species is a suitable species for studying cognitive abilities in fish. Specifically, the three main questions explored in this study were: (1) whether the two enrichment methods (habitat enrichment and social enrichment) can affect the growth performance of *S. schlegelii*; (2) whether the two enrichment methods can affect the cognitive ability of *S. schlegelii* and its molecular mechanism; and (3) determine the differences and interactions between the two enrichment methods, and provide a reference for the stock enhancement of *S. schlegelii*.

## 2. Materials and Methods

### 2.1. Experimental Design

Juvenile black rockfish, *S. schlegelii* (mean body weight: 13.85 ± 0.20 g, mean body length: 9.54 ± 0.15 cm), were obtained from Wendenhaihe Hatchery, a commercial hatchery in Weihai, Shandong, China. Before the experiment began, the juveniles were acclimated to the experimental conditions in an indoor tank (4 × 4 m) for 15 days. After acclimation, fish with similar initial body lengths and weights, free from body surface damage, were selected for the experiment. The experimental fish were reared in reinforced glass tanks (50 cm × 50 cm × 60 cm) with a continuous oxygen supply using gas stones to maintain the dissolved oxygen content of water (6.30 mgL^−1^). The water temperature gradually decreased from 19 °C to 13 °C during the experiment. The photoperiod followed the natural day–night cycle. The salinity was maintained between 28 and 30, and the water level was kept at 40 cm. The tanks were cleaned 3 times a week to avoid excessive interference with the fish, such as the production of oscillation when sucking the bottom for too long. The fish were fed with dry feed pellets (moisture, ≤10.0%; crude protein, ≥48.0%; crude lipid, ≥9.0%; crude ash, ≤17.0%; crude fiber, ≤2.0%; total phosphorus, 1.5–3.0%; and lysine, ≥2.5%; Kaido Brand, Santong Bioengineering Co. Ltd., Anqiu, China) at a quantity of 2.5% of their body weight every day during the rearing period.

The study was designed as a 2 × 2 factorial experiment with two factors: social enrichment (S) and habitat enrichment (H). For social enrichment, we used *Hexagrammos otakii*, which has similar habits to *S. schlegelii*, as the target of social enrichment. *H. otakii* were also hatched in the hatchery and had not been exposed to other species before the experiment began. In the natural environment, their habitats overlap and they sometimes have a competitive relationship. The factor was fixed with two levels: the presence (S+) or absence (S−) of *H. otakii* for social enrichment. For habitat enrichment, the factor was also fixed with two levels: structures and artificial plants (H+) or the absence of enrichment (H−). Therefore, four treatments (S−H−, S−H+, S+H−, and S+H+) were used in total. Each treatment group had three replicates, with eighteen fish in each replicate. The H+ group had structures that covered 50% of the tank’s bottom area, and the S+ group included fifteen *S. schlegelii* and three *H. otakii*. We selected this ratio based on their natural habits and to avoid increasing competition during the experiment. The rearing lasted for seven weeks from 8 September 2020 to 26 October 2020. After rearing, fifteen fish from each treatment group (five fish in each replicate) were selected for adaptation to the T-maze for three days. After adaptation, the T-maze experiment was carried out for a week. Finally, the fish were placed in MS-222 (200 mg/L) for 5 min to anesthetize them; then, their brain and visceral masses were sampled when they lost their balance and stored in liquid nitrogen rapidly.

### 2.2. Growth Measurements and Sample Collection

The experimental fish were weighed prior to their initial feeding. At the end of the rearing period, the fish were fasted for 24 h and weighed by tank. The specific growth rate, weight gain, and coefficient of body weight variation were measured.

Specific Growth Rate (%) = 100 × [ln (W_f_) − ln (W_i_)]/T

Weight Gain = (W_f_ − W_i_)/W_i_

Coefficient of Body Weight Variation (%) = 100 × SD/W_f_

where W_i_ (g) = mean initial body weight; W_f_ (g) = mean final body weight; T (d) = days of rearing; and SD = standard deviation of final body weight distribution. To carry out subsequent ELISA experiments, the fish were euthanized with MS222 and samples of the brain and visceral mass were collected. The telencephalon was then separated and stored in a liquid nitrogen tank for subsequent analysis.

### 2.3. T-Maze Test

**Figure 1:**
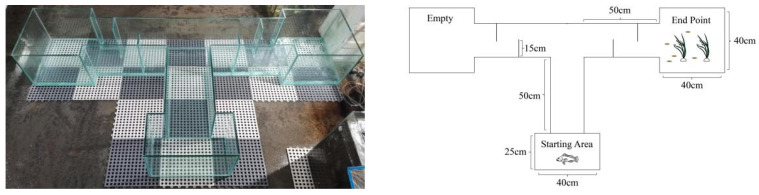
T-maze. Left: Profile Display; Right: Specification.

The T-maze device used in this study was designed based on a previous design [17], but with some improvements (Figure 1). The device was constructed from reinforced glass and divided into three chambers. The starting area consisted of a glass tank (40 cm × 25 cm), followed by a runway of 50 cm in length and 25 cm in width. Two identical runways measuring 50 cm in length and 25 cm in width were connected to the left and right sides of the runway, and two glass tanks (40 cm × 40 cm) were connected to each of the runways’ terminal areas. One of these end points contained an artificial habitat area enriched with plastic plants and physical structures for feeding training, while the other was an empty tank. To prevent fish from seeing directly into the end areas, two staggered frosted glass panels were added to each of the left and right runways. As the device was made of transparent glass, in order to prevent the fish from being affected by the surrounding environment, a camera was placed upon it and the researchers used remote monitoring to ensure quiet surroundings within 10 m during the experiment. The starting area also had a 2.8 cm diameter hole on its side and was connected to an outlet pipe, which allowed the water to be changed after each set of experiments. The water was still during the experiment.

After a 7-week rearing period, 60 fish (15 fish per treatment group, 5 fish per replicate) with vigor and no body surface damage were selected for the T-maze test. They were divided into twelve tanks (3 tanks per treatment group, 5 fish per tank) for temporary captivity according to their original order. To reduce stress and familiarize the experimental fish with the device, they were transferred to the device twice a day for 2 h in the morning and 2 h in the afternoon for a total of three days before the start of the T-maze test. We made sure that each fish had access to information about the availability of food in the enrichment zone while the fish acclimated to the device. At the end of each experiment, fish in different groups were put into separate tanks to avoid mixing with other fish.

The T-maze test lasted for a week, and the fish were fasted to maintain their food motivation. The experiment was conducted simultaneously for each treatment group. After a 15 min adaptation period, the fish were released into the starting area and the time taken to reach the habitat area for feeding or settling for at least 5 min was recorded. Individuals that failed to reach the habitat within 15 min were recorded as 900 s. The photoperiod followed a natural day−night cycle. In order to avoid the impact of light intensity and time change on each group, the measurement sequence was different every day.

The experiment was recorded using a Sony HDR-AS100V camera placed at a distance from the tank to avoid disturbing the fish.

### 2.4. Enzyme-Linked Immunosorbent Assay (ELISA)

The ELISA analysis was conducted to assess both growth-related factors (growth hormone: GH; insulin-like growth factor-1: IGF-1; and somatostatin: SS) and cognitive ability-related factors (brain-derived neurotrophic factor: BDNF; and nerve growth factor: NGF). GH and SS were measured in brain tissue, with six samples per treatment, while IGF-1 was measured in visceral mass, also with six samples per treatment. BDNF and NGF were measured in telencephalon tissue, with five samples per treatment. The samples were mixed with phosphate-buffered saline (PBS) at pH 7.4. The homogenates were centrifuged at 12,000× *g* for 10 min at 4 °C, and the supernatants were collected. The levels of GH, IGF-1, SS, BDNF, and NGF were measured using corresponding ELISA kits (Jianglai, Shanghai, China) according to the manufacturer’s instructions.

### 2.5. Data Analysis

All data were analyzed using SPSS 19.00 (IBM Corp., Armonk, NY, USA). In order to compare the effects of the two different enrichment methods on growth and cognitive indicators, the main and interaction effects of the two factors were tested by two-way ANOVA, followed by Duncan’s multiple-range post hoc test. Previously, data were tested for normality using the Shapiro–Wilk test and variance uniformity using the Levene test. In order to compare the differences in the learning speed of each group in the T-maze experiment, regression analysis was used and the slope of the fitted equation was used to represent the learning speed. A one-way ANOVA was also used to compare the differences in time spent among groups on the last day. OriginPro 2018C (OriginLab, Northampton, MA, USA) was used to perform linear fitting of the data of the T-maze test and was also used to plot the figures. All values in the text and figures were expressed as the mean ± S.E., and the differences were considered significant at *p* < 0.05.

## 3. Results

### 3.1. Growth Performance

**Figure 2:**
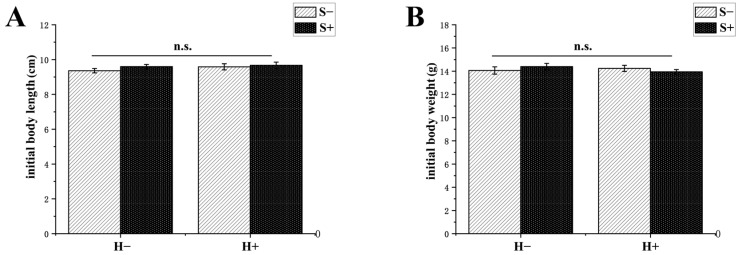

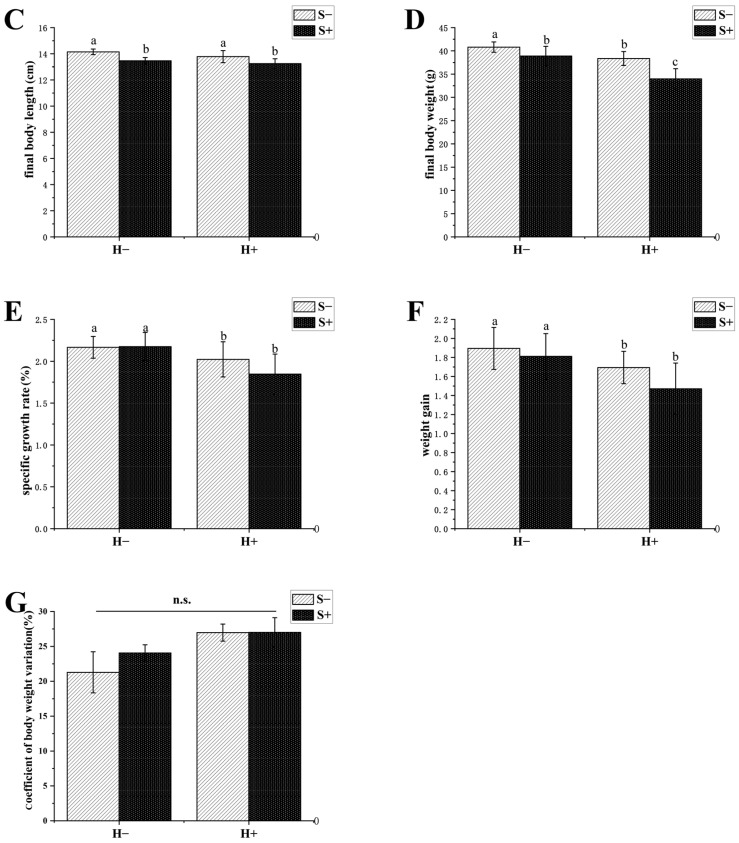
Growth performance of black rockfish *S. schlegelii* reared in different environments for seven weeks. (**A**) Initial body length; (**B**) Initial body weight; (**C**) Final body length; (**D**) Final body weight; (**E**) Specific growth rate; (**F**) Weight gain; (**G**) Coefficient of body weight variation. H−: no habitat enrichment; H+: habitat enrichment; S−: no social enrichment; S+: social enrichment. n.s.: *p* > 0.05. Different letters indicate significant differences among groups (*p* < 0.05). Data are presented as means ± S.E.

The two-way ANOVA revealed significant main or interaction effects of habitat enrichment and social enrichment on the final body length (habitat: *F*_1, 194_ = 2.073, *p* = 0.152; social: *F*_1, 194_ = 10.616, *p* = 0.001; and interaction: *F*_1, 194_ = 0.032, *p* = 0.857), final body weight (habitat: *F*_1, 194_ = 5.894, *p* = 0.016; social: *F*_1, 194_ = 4.205, *p* = 0.042; and interaction: *F*_1, 194_ = 1.237, *p* = 0.267), specific growth rate (habitat: *F*_1, 8_ = 12.174, *p* = 0.008; social: *F*_1, 8_ = 1.530, *p* = 0.251; and interaction: *F*_1, 8_ = 1.854, *p* = 0.210), and weight gain (habitat: *F*_1, 8_ = 11.185, *p* = 0.010; social: *F*_1, 8_ = 1.162, *p* = 0.312; and interaction: *F*_1, 8_ = 1.549, *p* = 0.248); however, there was no significant main or interaction effect on the initial body length (habitat: *F*_1, 194_ = 2.461, *p* = 0.118; social: *F*_1, 194_ = 2.861, *p* = 0.092; and interaction: *F*_1, 194_ = 0.692, *p* = 0.406), initial body weight (habitat: *F*_1, 194_ = 0.694, *p* = 0.406; social: *F*_1, 194_ = 1.516, *p* = 0.220; and interaction: *F*_1, 194_ < 0.001, *p* = 0.986), or coefficient of body weight variation (habitat: *F*_1, 8_ = 5.075, *p* = 0.054; social: *F*_1, 8_ = 0.537, *p* = 0.485; and interaction: *F*_1, 8_ = 0.513, *p* = 0.494, Table 1; Figure 2).

### 3.2. Results of the T-Maze Test

**Figure 3:**
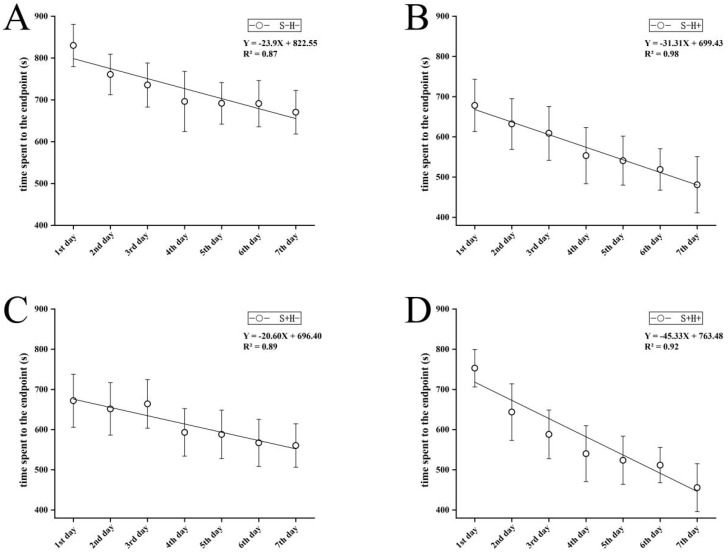
T-maze experiment: trend of time taken for fish to move from the starting area to the end point in four treatment groups ((**A**) control group, (**B**) habitat enrichment group, (**C**) social enrichment group, and (**D**) habitat and social enrichment group) in one week.

The variation trend of the time taken for each treatment group to find the end point of the T-maze within a week is shown in Figure 3. The mean time taken to reach the end point in the control group was significantly higher than in the other three treatment groups (one-way ANOVA, *F*_3, 24_ = 7.231, *p* < 0.05). The change in the time taken for each fish to find the reward zone during the experiment was representative of the difference in the learning speed of the different treatment groups in the T-maze, so we paid attention to the slope of the fitting line. By comparing the slope of the fitting line, except for social enrichment (S+H−, *k*: −20.60 ± 3.26), the time that fish in the habitat enrichment group (S−H+, *k*: −31.31 ± 2.03) took to find the end point decreased more quickly than that in the control group (S−H−, *k*: −23.90 ± 4.18), and the fish made the most significant progress when both enrichment methods were applied (S+H+, *k*: −45.33 ± 5.97). Comparing the differences in the time spent by fish to reach the end point on the last day among groups, that in the control group was significantly higher than that in the social enrichment group (*p* < 0.05), while those in the habitat enrichment group and the two-enrichment methods group were significantly lower than those in the social enrichment group (*p* < 0.05).

### 3.3. GH-IGF-1 Axis and SS

**Figure 4:**
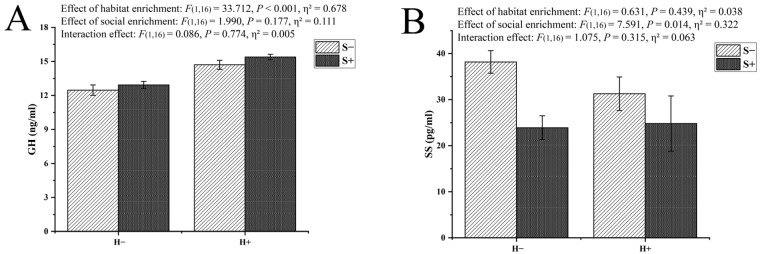

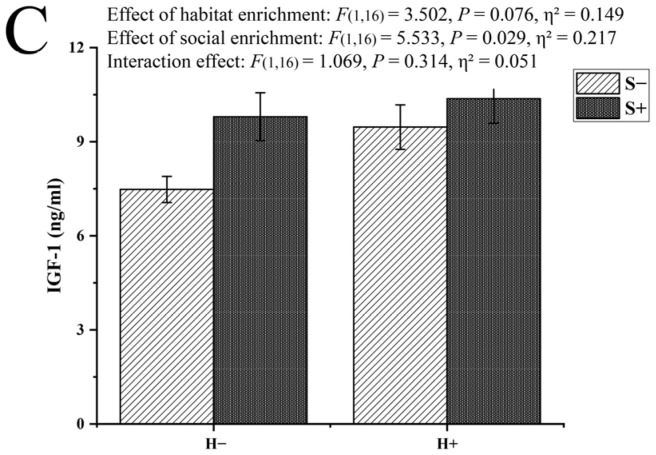
The levels of growth hormone (GH) and somatostatin (SS) in brain and insulin-like growth factor-1 (IGF-1) in visceral mass of black rockfish *S. schlegelii* reared under four different treatments for seven weeks. (**A**) GH level; (**B**) SS level; (**C**) IGF-1 level. Each horizontal coordinate (H−: no habitat enrichment, H+: habitat enrichment) corresponds to two treatments (S−: no social enrichment, S+: social enrichment), with a total of four treatments. The significance of the differences is indicated at the top of the bar chart, which was detected by Duncan’s multiple-range post hoc test (*p* < 0.05). Data are presented as the means ± S.E.

Significant main effects of habitat enrichment were detected in the GH measurement, while those of social enrichment were not. No interaction effect of the two factors was observed. However, it can be seen from the results that, when habitat enrichment and social enrichment were both provided, the level of GH was the highest (Figure 4A). 

In the measurement of SS, significant main effects of social enrichment were detected, while those of habitat enrichment were not. No interaction effect of the two factors was observed. As can be seen from the results, the brain SS level in the social enrichment group was significantly lower than that in the group without enrichment (Figure 4B). 

In the measurement of IGF-1, significant main effects of social enrichment were detected, while those of habitat enrichment were not. No interaction effect of the two factors was observed, but the results showed that the IGF-1 level in visceral mass was the highest when the two enrichment methods were both applied (Figure 4C).

### 3.4. Neurotrophic Factors

**Figure 5:**
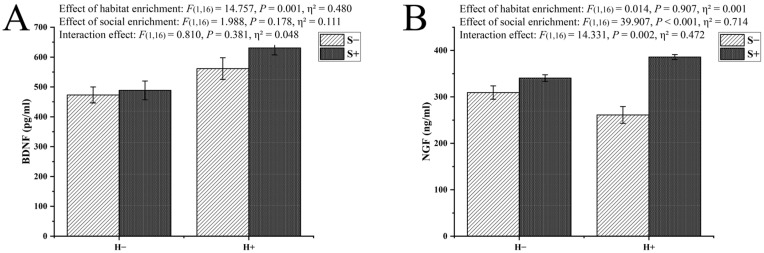
The levels of brain derived neurotrophic factor (BDNF) and nerve growth factor (NGF) in the telencephalon of black rockfish *S. schlegelii* reared in 4 different treatments for seven weeks. (**A**) BDNF level; (**B**) NGF level. Each horizontal coordinate (H−: no habitat enrichment, H+: habitat enrichment) corresponds to two treatments (S−: no social enrichment, S+: social enrichment), with a total of four treatments. The significance of the differences is indicated at the top of the bar chart, which was determined by Duncan’s multiple-range post hoc test (*p* < 0.05). Data are presented as the means ± S.E.

In the measurement of the BDNF level in the telencephalon, significant main effects of habitat enrichment were observed, while those of social enrichment were not. No interaction effect of the two factors was observed. However, it can be seen from the results that, when habitat enrichment and social enrichment both existed, the level of BDNF in the telencephalon was the highest (Figure 5A). 

In the measurement of the NGF level, significant main effects of social enrichment were observed, while those of habitat enrichment were not. In addition, an interaction effect of the two factors on the NGF level in the telencephalon was observed. As can be seen from the results, the level of NGF in the telencephalon was significantly higher than that in the control group or groups with single enrichment method when the two enrichment methods were both applied (Figure 5B).

## 4. Discussion

The main results of this study are as follows: (1) Both enrichment methods had an impact on the growth performance of juvenile *S. schlegelii*; however, their external performance (e.g., body length, body weight, specific growth performance, etc.; negative) differed from the measured levels of the three indicators regulating growth (GH, SS, and IGF-1; positive). (2) Both enrichment methods had an effect on the learning ability of juvenile *S. schlegelii*, as determined by the T-maze test, and increased the levels of BDNF and NGF, which are neurotrophic factors in the telencephalon related to cognitive ability. (3) Finally, a significant interaction was observed between the two enrichment methods on the NGF level in the telencephalon.

*S. schlegelii* tends to live in areas with rocky reefs and seagrass clumps in the wild, and adults have strong territorial behavior [30]. Juveniles incubated in the wild tend to be exposed to a complex environment from birth, which is not available for cultivated individuals. As environmental complexity affects brain morphology, it will invariably lead to related improvements in spatial cognition [31]. Their daily behavior, such as foraging, anti-predation, and navigation, all require complex memory and learning abilities regarding the spatial distribution of resources and shelters [32]. This is particularly important in aquatic environments, where resources may be diverted. Therefore, spatial cognition and continuous spatial information update are crucial for the survival of fish [33]. In contrast, the barren environment experienced by cultured juveniles tends to reduce their cognitive flexibility [34]. The results of our T-maze experiment also support that, compared with a barren environment, fish raised in a more complex environment performed better in the maze experiment, showing stronger learning and cognitive ability. Many studies on rodents have shown that barren environments can impair their neural development and plasticity [21]. In recent years, similar conclusions were reported in fish and great advances had been made in the study of fish brains [19]. It is known that the adult neurogenesis in fish brains is much higher than that in mammals [35,36], which suggests that the fish brain is a very plastic organ, and not just in the juvenile stage [9]. Most fish can adjust their physiology and behavior to adapt to the environment when faced with a complex and challenging environment [3,16]. As shown in this study, high spatial heterogeneity and the existence of species with similar living habits provided certain “challenges” to the *S. schlegelii*, thus enhancing their cognitive capacity and representing a greater learning ability. In addition, we observed that fish reared in an enriched environment also had higher cognitive flexibility, as they demonstrated in the T-maze. Such flexible adaptability is largely supported by cognition and neural plasticity [37,38]. The maze device was completely different from the rearing tank. After moving from the tank to the maze, the fish reared in an enriched environment showed stronger learning and adaptive capacity, indicating a faster ability to integrate new environmental cues, and thus greater cognitive flexibility. Therefore, we consider that there is a positive correlation between flexibility and learning ability. However, it may have a negative correlation with memory ability, which can be reflected in personality, behavior, and sex, and more attention needs to be paid to this in the future [39,40,41]. 

Previous studies have shown that many fishes have the capacity to learn and integrate information, while the processing of such information is a complex process [35]. The most important region in these complex neural processes of the brain is the dorsolateral telencephalon (DI) [42]. It is considered to be a functional homolog of the mammalian hippocampus [43]. The BDNF selected in this study is the most richly expressed member of the nerve growth factor family, which plays an important role in neural plasticity [21]. As a member of the neurotrophic factor family, another factor, NGF, selected in this study is also the earliest and most thoroughly studied one and plays an important role in regulating the development, differentiation, growth, regeneration, and expression of the functional characteristics of central and peripheral neurons [44]. Recent studies have also shown that environmental enrichment affects the expression of some cognitive-related factors and genes in fish brains [2,34,45]. The results of this study suggest that fish living in a complex environment for a long time have stronger learning and cognitive ability, and this situation may be related to the environmental “stimulation” of their brain. This stimulation promotes the up-regulation of BDNF and NGF and enhances neuroplasticity, which is essential for the survival of individuals for stock enhancement. However, only two members of the cognition-related neurotrophic factor family were selected in this study, and attention to other cognition-related factors should be considered in subsequent studies, including how increases in the BDNF and NGF levels lead to the improvement of cognitive ability, and whether other factors interact with them, as well as the primary and secondary status of different factors in the process of cognitive ability improvement. In addition, a more complex environment may cause a stronger stimulus and be conducive to increased cognitive flexibility. The result can provide a reference for improving the efficiency of stock enhancement for *S. schlegelii* [3,16].

Individual size is also one of the most basic key factors for the survival of fish [46]. In previous studies on salmonids, the size of individuals in smoltification affected their ability to adapt to seawater [47]. In this study, through the growth data analysis of juveniles after seven weeks of rearing, we found that habitat enrichment and social enrichment affected the body weight, body length, specific growth rate, and weight gain rate of juvenile *S. schlegelii*, and this effect was seemingly negative. This result seems to be different to those of previous studies [1,48]. Interestingly, through the follow-up of samples tested the three indicators associated with growth performance, we found that the GH and SS levels in the brain, and IGF-1 levels in the visceral mass of the experimental fish under different treatments had significant differences. Moreover, GH and IGF-1 in the enrichment treatment group were significantly higher than those in the control group, while SS was significantly lower than that in the control group, which was opposite to the growth performance. From the subsequent video analysis, we believe that the reasons for this phenomenon are as follows: Although the feeding amount of each tank was the same during the rearing period, due to the complex structure of the tank in the habitat enrichment treatment group, part of the bait fell into the structure and other places that were difficult for the experimental fish to find. As a result, the fish in the habitat enrichment treatment group ate less than the individuals in the other treatment group, resulting in a difference in growth. A similar phenomenon has been found in previous studies: starvation can significantly increase the plasma GH concentration in salmonids and pituitary GH mRNA in *Oreochromis* sp. and *Epinephelus coioides* [49,50]. Additionally, starved fish refeeding can lead to a rise in the hepatic IGF-I concentration and IGF-1 mRNA [51]. In addition to affecting growth performance, starvation can also affect the behavior of fish, and even briefly affect their personality. Personality includes courage, exploratory, aggressive, etc. Changes in the external environment can sometimes affect the differences in personality indicators between individuals in a population, resulting in differences in behavior, which may have an impact on maze experiments [28,52]. On the other hand, as the habitat range and living habits of *H. otakii* and *S. schlegelii* are relatively close, the presence of *H. otakii* may cause “suppression” to *S. schlegelii*. Previous studies have shown that competition and pressure can affect the physiological state and personality behavior of fish, but we had not explored whether this “suppression” also affects the behavior of the experimental fish. Therefore, follow-up studies should pay more attention to the relationship among personality behavior, learning cognitive ability, and stress [53]. As the hormone level regulating growth in the enrichment group was higher than that in the control group, we believed that the experimental fish would show compensatory growth in the subsequent process under this background [54,55,56,57]. In order to avoid this phenomenon and improve the utilization rate of food, attention should be paid to the location of the structure and the selection of species in follow-up studies of environmental enrichment, so as to avoid reducing food utilization. Additionally, the selection of food type and feeding method can also be improved, such as a small amount and high frequency of feeding to avoid waste of food and an effect on the growth of fish.

In general, compared with the control group, the levels of BDNF and NGF were up-regulated in both the habitat and social enrichment groups, and there was a significant interaction effect between the two enrichment methods on NGF. This may have been caused by the stimulation of cognitive processes, such as learning and memory, due to the complexity of the living environment [2,8]. At the same time, the results of the behavioral evaluation were consistent with them. In the T-maze experiment, both the habitat and social enrichment groups performed better in the T-maze, and the time needed to find the end point within 7 days was shorter than that of the control. We considered that the two enrichment methods improved the learning and cognitive ability of the experimental fish.

## 5. Conclusions

In this study, we discovered that seven weeks of rearing with two enrichment methods can improve the cognitive and learning ability of juvenile *S. schlegelii* about 9.5 cm in length, which provides a favorable reference for stock enhancement. However, the enrichment method needs to be improved, such as the placement of structures, to avoid affecting the feeding of the fish. The goal of stock enhancement should be to rear juveniles that resemble the wild population in morphology, behavior, and physiology. In addition, it is necessary to measure a wider range of cognitive ability-related indicators and explore the relationship between different indicators and a whether common effect existed, so as to explore the mechanism more deeply. Considering other factors affecting the survival of juveniles after release, further investigation is needed to determine the field survival rate, especially detection and tracking after release.

## Figures and Tables

**Table 1:** Results of two-way ANOVA on growth performance indicators among different treatment groups. *p* < 0.05 indicates that the enrichment method had a significant effect on the indicator. η^2^ represent the effect size.

Indicators	Enrichment Methods	*F*	*p*	η^2^
Initial body length	Habitat	2.461	0.118	0.013
	Social	2.861	0.092	0.015
	Interaction	0.692	0.406	0.004
Initial body weight	Habitat	0.694	0.406	0.004
	Social	1.516	0.220	0.008
	Interaction	<0.001	0.986	0.001
Final body length	Habitat	2.073	0.152	0.011
	Social	10.616	0.001	0.052
	Interaction	0.032	0.857	0.001
Final body weight	Habitat	5.894	0.016	0.029
	Social	0.016	0.042	0.021
	Interaction	1.237	0.267	0.006
Specific growth rate	Habitat	12.174	0.008	0.603
	Social	1.530	0.251	0.161
	Interaction	1.854	0.210	0.188
Weight gain	Habitat	11.185	0.010	0.583
	Social	1.162	0.312	0.127
	Interaction	1.549	0.248	0.162
Coefficient of body weight variation	Habitat	5.075	0.054	0.388
	Social	0.537	0.485	0.063
	Interaction	0.513	0.494	0.060

## Data Availability

The partial data analyzed for this study are available from the corresponding author upon reasonable request.
